# A multimodal blended learning curriculum for ultrasound-guided diagnostics and needle navigation: development and early implementation in an Indian tertiary care setting

**DOI:** 10.3389/fmed.2026.1697471

**Published:** 2026-05-08

**Authors:** Rashmi Ramachandran, Kritika Sharma, Nishkarsh Gupta, Abhishek Nagarajappa, Subodh Kumar, Ambuj Roy

**Affiliations:** 1 Department of Anaesthesiology, Pain Medicine and Critical Care, All India Institute of Medical Sciences, New Delhi, India; 2 Skills, E-Learning and Telemedicine Facility, All India Institute of Medical Sciences, New Delhi, India; 3 Department of Onco-anaesthesia and Palliative Medicine, Dr BRA IRCH, All India Institute of Medical Sciences, New Delhi, India; 4 Department of Cardiology, All India Institute of Medical Sciences, New Delhi, India

**Keywords:** asynchronous e-learning, blended learning, needle navigation, simulation, ultrasound-guided diagnostic

## Abstract

**Introduction:**

Ultrasound-guided diagnostic and needle navigation procedures are integral to modern medical practice, yet structured competency-based training remains limited within the Indian medical education system. This paper describes the development and early implementation of a simulation-enhanced blended learning curriculum delivered through the institutional Learning Management System (SARAL) at AIIMS, New Delhi.

**Methods:**

The program combined asynchronous online modules with simulation-based and supervised hands-on training. Formative evaluation was conducted via a cross-sectional survey of faculty involved in programme delivery (*n* = 12), assessing course design, pedagogy, perceived learner applicability, and feasibility using Likert-scale items and open-ended feedback.

**Results:**

Formative feedback from 12 expert faculty involved in programme design and delivery indicated strong agreement on the curriculum's relevance, coherence, and clinical applicability, as well as its feasibility for broader implementation. Key recommendations included increasing hands-on exposure, reducing group sizes, and integrating advanced digital learning tools.

**Conclusions:**

These findings reflect early expert appraisal of a curriculum in its initial implementation phase and do not represent learner-level outcomes. Formal evaluation of educational impact is planned in future phases. The report provides a context-specific account of curriculum development and implementation that may inform similar efforts in resource-constrained settings like India.

## 1 Introduction

Ultrasound (US)-guided diagnostic and needle navigation procedures are integral to modern medical practice, especially in emergency, anaesthesiology, and critical care settings (1). Building on this diagnostic capability, US-guided interventions such as needling enables precise real-time visualization of needle trajectory, significantly enhancing the safety, accuracy, and success of various interventional procedures, including biopsies, fluid aspiration, targeted drug delivery, nerve blocks, and vascular access (2, 3). These advantages reduce reliance on more invasive surgical interventions, support real-time clinical decision-making, and contribute to improved patient outcomes, solidifying the role of US as a critical tool in contemporary medical practice (4).

A significant barrier to the consistent and safe application of US-guided procedures is the lack of structured, effective education and training programs (5). As clinical reliance on US-guided diagnostics and techniques grows, particularly for procedures requiring high precision, there is a critical need for educational strategies that are scalable, resource-efficient, and pedagogically effective (6). Such models will facilitate the creation of engaging, learner-centered experiences that promote skill acquisition and retention. Interventions in this area have been successfully implemented across multiple medical specialties, demonstrating learner engagement, skill acquisition, and knowledge retention in US-guided diagnostic and needle navigation procedural training (7).

This curriculum report describes the development and early implementation of a structured, competency-based blended learning curriculum for ultrasound-guided diagnostics and needle navigation in a tertiary care academic setting in India, designed to address well-recognized barriers and systemic gaps that have limited the integration of structured ultrasound training into medical education. At the time of writing, the curriculum is in its initial implementation phase, with faculty modules deployed and learner enrolment underway; therefore, the paper focuses on the contextual rationale, curriculum design, and implementation framework, supplemented by formative insights from expert faculty to support iterative refinement rather than to report educational outcomes. A formal evaluation of learner-level outcomes, including skill acquisition and clinical performance, is planned for a subsequent phase, and by documenting this early experience, providing practical framework for scalable ultrasound training, particularly in resource-constrained settings where financial and logistical barriers often limit access to structured, competency-based education.

## 2 Challenges in acquiring proficiency in US-guided diagnostics and needle navigation techniques

Mastering US-guided procedures requires the simultaneous development of cognitive, visual-spatial, and psychomotor skills, posing significant challenges (8). The procedures require a high level of hand and eye coordination to precisely guide instruments or probes while simultaneously observing real-time imaging. Mastery of these skills is critical for ensuring both the accuracy and safety of the intervention. Learners often struggle with anatomical recognition, image optimization, maintaining needle visibility throughout the procedure, and mastering “knobology”, the manipulation of US machine controls to optimize imaging for accurate guidance (9). The steep learning curve is compounded by limited access to expert supervision, variable quality of instructional materials, and inconsistent opportunities for hands-on practice (10). Operating US equipment and interpreting real-time images demands extensive practice and training. For novices, visualizing anatomy in a 2D grayscale image and converting it into a 3D representation can be particularly challenging, as it requires advanced spatial awareness and a deep understanding of anatomical structures (11).

Furthermore, hands-on experience may be scarce, particularly for complex or low-frequency procedures, making it challenging to achieve proficiency (12, 13). These challenges highlight the significant need for structured, evidence-based educational interventions to support the development of competency in US-guided techniques. The use of US during procedures may initially increase procedure time, which can lead to reluctance among practitioners to adopt the technique (14). These challenges can be more precisely categorized into practical challenges and organizational challenges, which involve structural and administrative factors (including curriculum integration, institutional support, and scheduling constraints) (6). To overcome these challenges, structured training programs, simulation-based practice, and mentorship from experienced practitioners are crucial for developing proficiency.

## 3 Blended learning as a strategy to address training challenges in US-guided diagnostics and needle navigation procedures

Blended learning, which integrates asynchronous digital instruction with structured face-to-face training, was adopted as it offers a practical and pedagogically aligned approach for ultrasound-guided procedural education. Ultrasound-guided diagnostics and needle navigation require the coordinated development of conceptual understanding, image interpretation, and hands-on procedural skills, which are difficult to address through traditional training models alone (10).

The blended learning approach was selected to address key barriers identified in ultrasound training, including limited access to expert faculty, variability in clinical exposure, and restricted opportunities for repeated hands-on practice. Asynchronous online modules will allow learners to acquire foundational knowledge, including ultrasound physics, image interpretation, and procedural principles, at their own pace. This is intended to support more consistent baseline level of preparation and understanding prior to in-person training.

Face-to-face components, including simulation-based sessions and supervised workshops, will be used to reinforce psychomotor skills, provide real-time feedback, and facilitate deliberate practice in a controlled learning environment. (15). This structure reflects a pragmatic design choice, in which different instructional modalities are aligned with the types of competencies being addressed, rather than a formal application of a specific theoretical framework.

## 4 Current status of POCUS and US-guided procedures in Indian medical education

In India, the integration of POCUS and US-guided procedural skills, such as needle navigation, into medical training programs remains limited (16). Postgraduate programs in Emergency Medicine (EM) in North America routinely incorporate POCUS as a core component of clinical decision-making and procedural guidance. Indian medical curricula, particularly at the undergraduate MBBS level, do not currently include structured, competency-based training in these areas. The National Medical Commission (NMC), a statutory body in India that regulates medical education, medical professionals, medical institutions, and medical research, does not mandate formal US training as part of the undergraduate medical curriculum. Exposure to diagnostic US is typically limited to radiology postings, with minimal hands-on experience (17).

Furthermore, regulatory restrictions on the use of US by non-radiologists, influenced by the Pre-Conception and Pre-Natal Diagnostic Techniques (PC & PNDT) Act, 1994, which is an Indian law that aims to prohibit sex-selective abortions and regulate prenatal diagnostic techniques to prevent female feticide, present additional barriers to the integration of POCUS into routine clinical practice and training (18). The PC & PNDT Act mandates that only individuals with an MD in Radiology or those who have completed a six-month certified training program are authorized to perform US examinations, particularly in obstetric settings (19). This regulation aims to prevent misuse of US for sex determination, but inadvertently limits the broader application of POCUS by non-radiologist clinicians (20).

At the postgraduate level, some emergency medicine and critical care programs have begun to introduce focused US modules; however, there is significant variability in access to trained faculty, equipment, and curriculum standardization. Workshops and conferences have started offering POCUS training sessions, reflecting a growing interest in incorporating these skills into clinical practice. These systemic limitations have resulted in a fragmented approach to US education, with few centers offering structured, simulation-based, or blended learning pathways for procedural skill acquisition, including needle navigation. The lack of uniform training standards and regulatory constraints continues to pose challenges to the widespread adoption of POCUS in Indian medical education (21, 22).

## 5 SARAL (student advanced resources and learning) enabled blended training in US-guided diagnostics and needle navigation

The platform enables educators to curate and disseminate instructional materials such as pre-recorded lectures, interactive modules, and reading assignments. SARAL also facilitates progress tracking, feedback provision during in-person sessions, and the coordination of small-group simulation-based hands-on training.

There exists a significant and well-documented need for structured training in US-guided diagnostic and interventional skills among in-service medical professionals in India (23, 24). Preferences for flexible, simulation-based, and hands-on modalities indicate a strong inclination toward blended learning formats. Furthermore, there is a clear demand for formal recognition of skills through certifications aligned with institutional and regulatory standards (25). These insights collectively advised the development of a tailored, expert-led blended learning model designed to bridge the identified gaps and support scalable, practice-oriented training in US-guided needling procedures.

**Figure 1:**
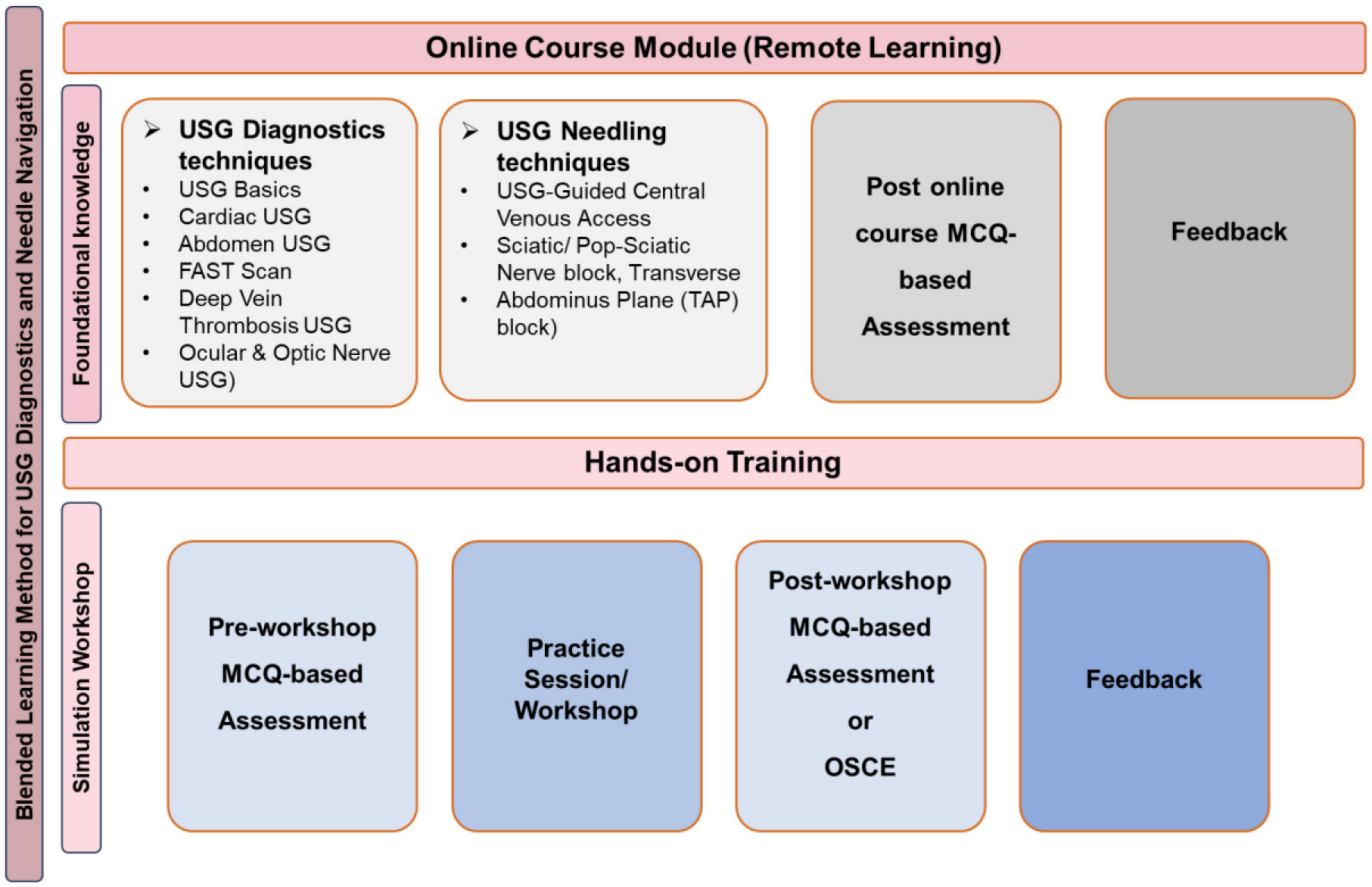
Course overview for continuing professional development of healthcare professionals in US-guided diagnostics and needle navigation.

Guided by the insights derived, a panel of 21expert faculty members collaboratively designed a blended learning model tailored to US-guided diagnostics and needling competencies using SARAL is the integrated Learning Management System utilized at AIIMS, New Delhi as depicted in Figure 1.

Both US-guided diagnostics and needling courses follow a structured blended learning model combining online instruction with hands-on training. The diagnostics course begins with asynchronous modules comprising PDFs, video lectures, and MCQ-based assessments covering core concepts such as equipment handling, probe orientation, and image interpretation across applications including echocardiography, lung, abdominal, FAST, DVT, and ocular ultrasound. This is followed by small-group, faculty-supervised hands-on sessions incorporating pre- and post-assessments. The needling course builds on this foundation through targeted online modules with integrated MCQs, followed by practical workshops that include pre- and post-workshop Objective Structured Practical Examinations (OSPEs), structured skill training, and formative feedback. Key procedural competencies include US-guided central venous access, transversus abdominis plane (TAP) block, and sciatic nerve block, emphasizing safe and effective clinical application.

Our SARAL platform has enabled a blended learning model for US-guided diagnostics and needle navigation courses, which constitutes a pivotal advancement within the framework of Indian medical education. By seamlessly integrating asynchronous e-learning modules with structured, hands-on skill development, this model addresses the pressing need for scalable, competency-driven training in the US, a domain of growing significance in both diagnostic and interventional practice. Given the limitations inherent in conventional postgraduate medical curricula, particularly concerning faculty availability and variability in clinical exposure, SARAL offers a standardized, accessible, and flexible pedagogical platform. It facilitates the acquisition of core theoretical competencies through self-paced learning while preserving in-person training time for supervised procedural practice and expert-guided skill refinement.

## 6 Expert faculty insights on the evaluation framework, curriculum design, and scalable implementation of US-guided diagnostic and needling training

To support iterative refinement and quality assurance, structured formative feedback was obtained from expert faculty involved in the design and delivery of the curriculum. The purpose of this exercise was not to evaluate learner outcomes, but to capture faculty perspectives on curriculum design, pedagogical alignment, and the feasibility of broader implementation within medical education curricula. A structured evaluation framework was developed to obtain expert feedback across four domains: (i) Course content and design, (ii) Pedagogical approach, (iii) Perceived learner engagement and applicability to clinical practice, based on faculty observations, and (iv) Programme feasibility and future implementation, using open-ended responses to capture suggestions for scalability, integration, and further development. The evaluation was administered via a survey (Google form) to obtain feedback from expert faculty.

A total of 12 faculty members who were directly involved in the design and delivery of the curriculum participated in this formative feedback exercise. As shown in Table 1, the composition reflects a group of domain experts with diverse clinical exposure to ultrasound-guided practices across specialties. Their perspectives therefore provide insight into the perceived relevance, structure, and feasibility of the curriculum from an implementation standpoint. At the same time, as contributors to the programme's design and delivery, their feedback should be interpreted as informed insider appraisal intended to guide iterative refinement, rather than as independent evaluation of programme effectiveness.

**Table 1:** Demographic characteristics of faculty (*N* = 12).

Variable	Category	Frequency (*n*)	Percentage (%)
Age group	< 40 years	8	66.7
	≥40 years	4	33.3
Gender	Male	9	75.0
	Female	3	25.0
Designation	Assistant professor	6	50.0
	Associate professor	3	25.0
	Professor	3	25.0
Department	Anaesthesiology and pain medicine (all subspecialties)	5	41.7
	Cardiology	3	25.0
	Pulmonary/critical care	1	8.3
	Cardiac radiology	1	8.3
	Onco-anesthesiology and palliative medicine	1	8.3
	Radiology (subspecialty)	1	8.3
Experience	< 5 years	3	25.0
	5–10 years	5	41.7
	>10 years	4	33.3

**Figure 2:**
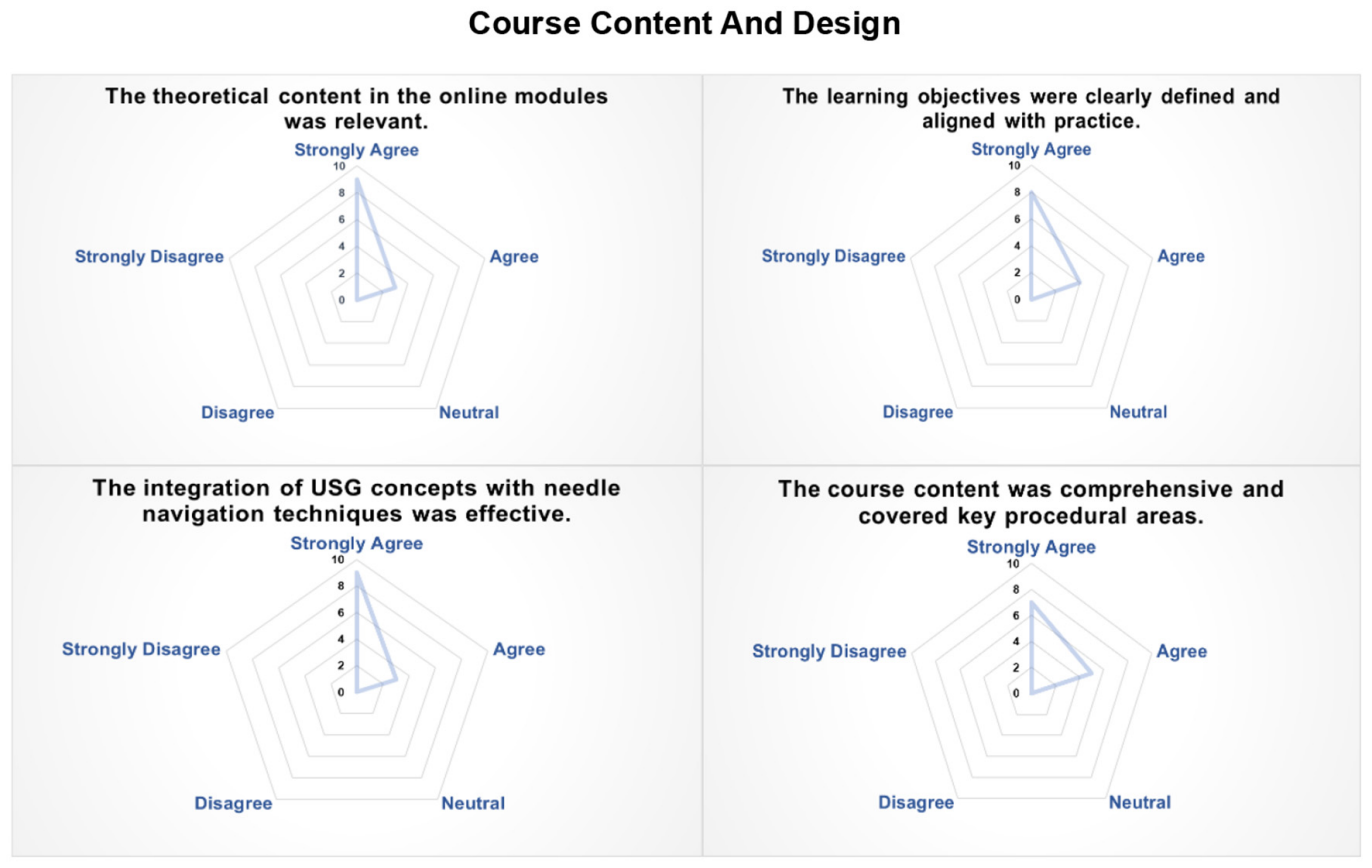
Radar plot illustrating expert faculty's responses on a 5-point Likert scale regarding the US training program's course content and design.

Expert faculty provided ratings on course content and design using 5-point likert-scale (Figure 2). Overall, respondents expressed uniformly positive perceptions across all items. The theoretical content was considered clinically relevant (9 strongly agreed; 3 agreed), and the learning objectives were viewed as clearly aligned with practical competencies that are expected to support real-world application (8 strongly agreed; 4 agreed). The integration of ultrasound principles with needle navigation techniques was positively appraised (9 strongly agreed; 3 agreed). Similarly, the content was perceived as comprehensive in covering key procedural domains (7 strongly agreed; 5 agreed). No neutral or negative responses were recorded, suggesting consistent agreement among participating faculty regarding the relevance, clarity, and instructional design of the curriculum.

**Figure 3:**
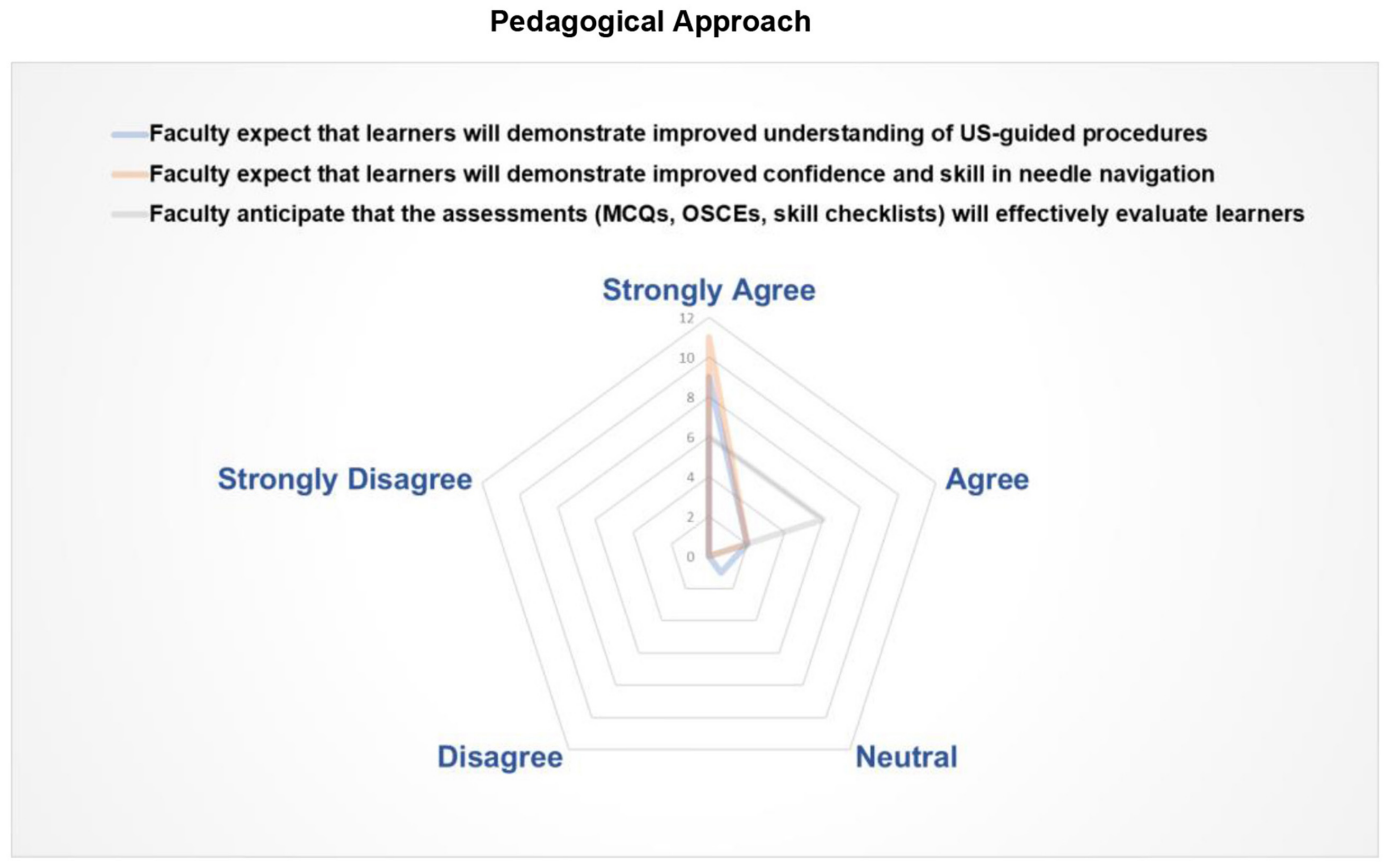
Radar plot representing expert faculty ratings (5-point Likert scale) of the programme's pedagogical design, which incorporates integrated blended learning, simulation-based training, and aligned assessments to enhance cognitive and procedural skills in US-guided techniques. Items reflect faculty expectations regarding the potential of these components to support cognitive and procedural skill development of learners in US-guided techniques.

Expert faculty perceptions of the pedagogical approach are summarized in Figure 3. It suggested that the curriculum design is expected to support learners' understanding of ultrasound-guided procedures (9 strongly agreed; 2 agreed; 1 neutral) and has the potential to support confidence and technical proficiency in needle navigation (11 strongly agreed; 1 agreed), although these views are not based on direct learner outcome data. All faculty considered the assessment methods (MCQs, OSCEs, and skill checklists) are appropriate for evaluating performance within this framework (6 strongly agreed; 6 agreed). Overall, this expert faculty perception between teaching, practice, and assessment indicates a structured and pedagogically consistent curriculum framework.

**Figure 4:**
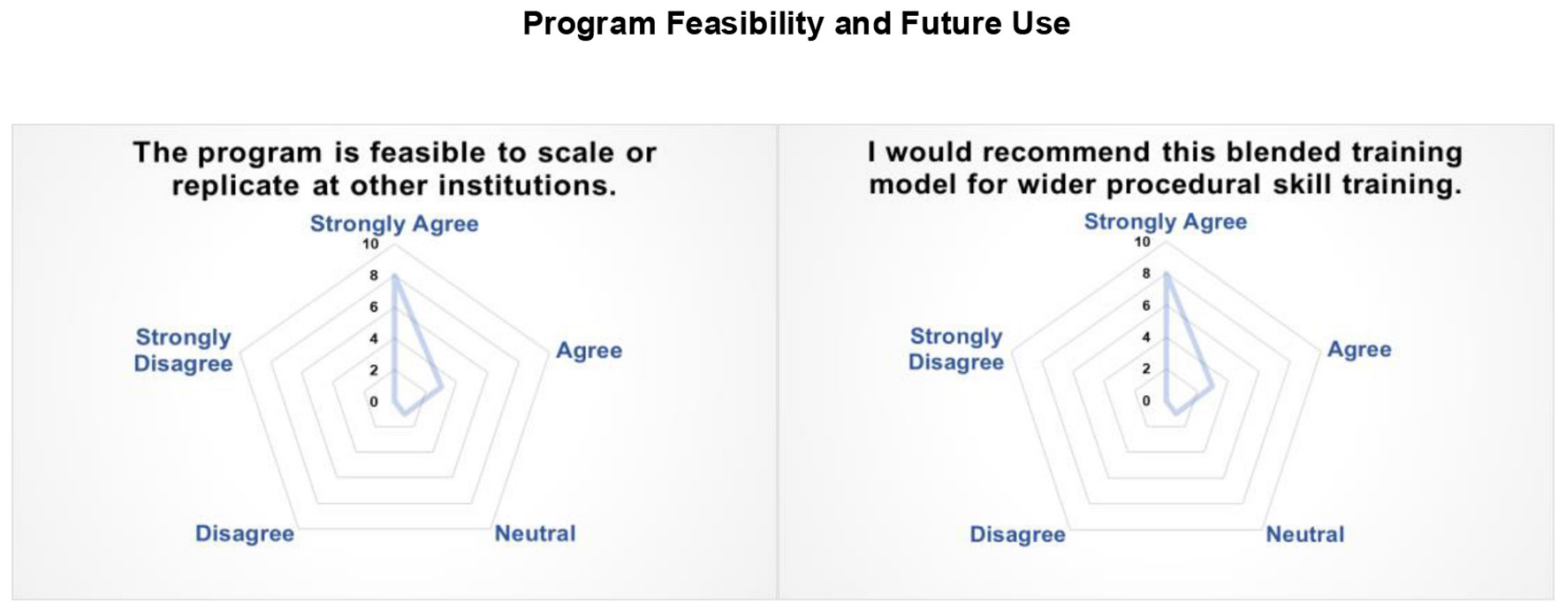
Radar plot illustrating expert faculty ratings (5-point Likert scale) regarding the perceived scalability, institutional adoption, and broader applicability of the blended training model for procedural skill development.

Faculty perceptions of programme feasibility and future applicability revealed strong support for broader implementation and potential scale-up of the blended training model (Figure 4). Eleven of the expert faculty agreed or strongly agreed regarding the anticipated scalability and transferability of the programme across institutional contexts, with one neutral response and no dissent; a similar level of endorsement was noted for its prospective application in skills training.

Overall, feedback across domains of curriculum design, pedagogical approach, and implementation feasibility suggests that the programme is well-positioned for implementation. The curriculum is expected to provide a comprehensive, contextually relevant, and coherently structured framework to support procedural skill acquisition. The blended model integrating asynchronous learning, simulation-based training, and aligned assessment strategies is anticipated to facilitate cognitive and technical skill development, although its educational impact will require evaluation through future learner-level outcomes.

Recommendations for subsequent iterations are directed toward strengthening implementation and optimizing educational delivery. Faculty emphasized the need to augment teaching capacity to improve supervision, reduce learner group sizes to enable deliberate practice, and incorporate mandatory pre-course engagement with asynchronous modules to ensure baseline preparedness. The introduction of workplace-based assessments is proposed to support the translation of simulated competencies into clinical practice. Additional recommendations include the integration of advanced instructional resources, such as serious gaming tools, intraoperative procedural videos, virtual case-based discussions, and 3D anatomical visualizations. The inclusion of curated open-access content, cadaveric workshops, and advanced modular extensions is also suggested to support progressive skill development and future specialization pathways.

## 7 Reflections on curriculum implementation and limitations

Despite the diagnostic and therapeutic utility, US-guidance remains underrepresented in traditional training programs across India (17). This curriculum report describes the implementation of a blended learning model integrating asynchronous modules, simulation-based training, and supervised hands-on workshops to address these gaps. This approach integrates asynchronous online modules, simulation-based training, and supervised hands-on workshops will enhance both theoretical knowledge and procedural skills. Simulation forms the cornerstone of technical training, offering a low-risk environment for repeated practice, real-time feedback, and performance assessment. (26).

There is robust evidence in the literature on the effectiveness of structured blended learning US training for both pre-service and in-service healthcare professionals. In Denmark, a hybrid effectiveness implementation study involving 18 office-based general practitioners demonstrated that a 3-month tailored POCUS program significantly boosted scanning competence, scores rose from 68.9% to 82.3% post-training to 80.9% to 92.6% at 3-month follow-up, with sustained use in daily practice (27). Jonck et al. conducted a prospective study with 72 paramedics undergoing a blended course combining online theory and in-person hands-on sessions. Their theoretical scores improved significantly between pre- and post-module assessments, and by course end, participants achieved high practical competence (≈87% on healthy subjects; ~84% on simulators), comparable to medical students and physicians (28). These data from prior studies suggests that immersive, competency-based US-guided blended training, with simulated or clinical exposure, leads to marked improvements in skill, confidence, and clinical utility, providing a strong comparative framework for implementing and assessing similar educational models in India.

The implementation of the SARAL-enabled curriculum demonstrates the feasibility of a scalable, LMS-supported model within a tertiary care setting, contingent on institutional readiness, faculty engagement, and access to appropriate infrastructure. The prevalence of the perceived barriers to its implementation may vary across different regions in India. Understanding these barriers is a crucial first step in designing effective, context-specific strategies to address them.

A key methodological limitation of this study is that the evaluation is based entirely on feedback from faculty who were directly involved in the design and delivery of the programme. This introduces a high likelihood of positive bias, as responders are evaluating a curriculum in which they have intellectual and professional investment. The uniformly positive responses observed across Likert-scale items are therefore not interpreted as evidence of programme effectiveness, but rather as indicative of their self-reported confidence in alignment and perceived coherence among programme developers. Such insider appraisal is valuable for formative refinement but cannot substitute for independent evaluation or provide evidence of educational impact. Future studies will require external evaluation and learner-level outcome data to establish effectiveness.

Successful and reliable implementation of US-guided diagnostics and needle navigation training requires a robust digital infrastructure, consistent learner engagement, and sustained faculty involvement. However, technical challenges such as limited internet connectivity, inadequate hardware, and issues related to platform usability can significantly impact the learning experience, particularly in peripheral or resource-limited institutions. Additionally, ensuring the quality and fidelity of simulation-based training across diverse training sites poses further challenges (6).

## 8 Summary

This curriculum report describes the development and early implementation of LMS (SARAL) enabled blended learning program for US-guided diagnostics and needle navigation within an academic tertiary care setting. The model integrates asynchronous learning, simulation-based training, and structured hands-on practice within a competency-oriented framework designed to address recognized gaps in procedural training in India. Given the multiple skill components involved, including image interpretation and procedural coordination within a unified curriculum, the model is designed to support the competencies required for safe and effective clinical practice. Future work will focus on systematic evaluation of learner performance, skill acquisition, and clinical impact to more robustly assess the educational value of this blended learning approach. As the healthcare landscape continues to evolve, integrating scalable, competency-based training models like this will be essential for preparing clinicians to meet the contemporary demands of patient care, especially in India.

## Data Availability

The original contributions presented in the study are included in the article/supplementary material, further inquiries can be directed to the corresponding author.
